# What About Well‐Being? Measuring What We Really Care About in Sexual and Reproductive Health

**DOI:** 10.1111/sifp.70022

**Published:** 2025-06-09

**Authors:** Christine Dehlendorf, Shashi Sarnaik, April J. Bell, Alyssa Lindsey, Jamie Hart, Sheila Desai, Bilgissou Balde, Christine Galavotti, Ewenat Gebrehanna, Jennifer Hall, Renu Khanna, Anne Philpott, Lore Remmerie, Niranjan Saggurti, Zeba A. Sathar, Miranda van Reeuwijk, Vicky Boydell

## Abstract

The well‐being of individuals and communities is increasingly recognized as a core objective of economic and global development policies and programs. However, existing measures of well‐being neglect sexual and reproductive experiences, which are core dimensions of people's lives. While there has been increasing attention to the concept of sexual well‐being, measures of sexual and reproductive health are predominantly deficit‐based and ignore whether people are having positive experiences. To consider the development of a measure of sexual and reproductive well‐being, a multidisciplinary and geographically diverse group of experts was convened. Outcomes of this meeting included endorsement of a draft definition of sexual and reproductive well‐being, demonstrated enthusiasm and commitment to the development of a measure capturing this construct, and delineation of core considerations in the measure development process. These included considering the diversity of normative and political contexts around sexuality and reproduction, and the critical nature of meaningful community engagement when developing this measure. A pathway for measuring development was defined, with the goal of creating a concise measure assessing people's holistic experiences of sexuality and reproduction that can draw attention to and monitor the extent to which people are having the sexual and reproductive lives they wish to have.

## INTRODUCTION

Sexuality and reproduction are core aspects of the human experience, and the extent to which people can achieve healthy and fulfilled sexual and reproductive lives influences people's mental, emotional, and physical health throughout the course of their lives. However, in public health and global development discourse and data collection, sexuality and reproduction are viewed through the lens of population‐level rates of disease and adverse health outcomes, such as sexually transmitted diseases, unintended pregnancy, and maternal mortality (Bingenheimer et al. 2023). These outcomes are grounded in a deficit framing of sex, sexuality, and reproduction, with an emphasis on what we are trying to avoid while overlooking equally important facets such as our hopes, preferences, satisfaction, and joy (Van Bortel et al. 2019; Kågesten and van Reeuwijk 2021; Philpott et al. 2021). This unitary focus on measuring and addressing adverse outcomes contributes to the stigmatization of populations at higher risk for these outcomes while failing to consider whether or not people can achieve positive sexual and reproductive experiences (Suvirya et al. 2018; Ma et al. 2022; Logie et al. 2024; Zia et al. 2024). There is, therefore, a critical need to advance person‐centered rights and equity‐based measures that provide an asset‐based lens on sexuality and reproduction. This is particularly important in the context of the increasing traction and influence of anti‐rights and anti‐gender movements (Murray 2022; Gilby and Koivusalo 2020). Transforming and innovating measures to be values‐aligned and supportive of human rights can provide a valuable tool to push back on these efforts to undermine sexual and reproductive rights and equity across the globe.

The recent special issue on *Indicators in Sexual and Reproductive Health* provided a thoughtful critique of deficit‐based approaches, with papers that delineated problems with measures (e.g., total fertility rates, contraceptive prevalence rates, unmet need) traditionally used in the field of sexual and reproductive health due to their prioritization of demographic, health, and behavioral outcomes to the detriment of people's preferences (Bingenheimer et al. 2023; Speizer, Bremner, and Farid 2022). The special issue also highlighted exciting innovations in measures of individuals’ preferences and experiences (Burke and Potter 2023; Holt et al. 2023a), focusing on people's interactions and experiences with healthcare. The special issue and related ongoing discussions in sexual and reproductive health sectors are critical steps forward in disrupting traditional approaches to measurement and moving towards centering individuals and their experiences.

An additional and untapped opportunity to further advance this transformation of sexual and reproductive health measures is a focus on the ultimate outcome of interest: whether people are living their optimal sexual and reproductive lives. Looking towards a measure of such an aspirational outcome is consistent with the definition of reproductive health included in the International Conference on Population and Human Development (ICPD) Programme of 1994 (UNPF 1994), which stated that

Reproductive health is a state of complete physical, mental and social well‐being and not merely the absence of disease or infirmity, in all matters relating to the reproductive system and to its functions and processes. Reproductive health therefore implies that people are able to have a satisfying and safe sex life and that they have the capability to reproduce and have the freedom if, and when, and how often to do so.

This vision of sexual and reproductive health from a holistic and positively‐framed lens has been reflected in publications and statements since this ICPD statement, such as in the World Health Organization's definition of sexual and reproductive health (WHO 2006). More recently, leaders in global sexual and reproductive health endorsed an aspirational vision in the Guttmacher–Lancet Commission by stating that “a positive approach to sexuality and reproduction should recognize the part played by pleasurable sexual relationships, trust, and communication in the promotion of self‐esteem and overall wellbeing” (Starrs et al. 2018).

A common thread in these documents is the reference to well‐being, which describes what we should aspire to in supporting people's sexual and reproductive lives. This term draws on a robust and growing emphasis on well‐being in political and economic spheres as the desired end goal that governments should strive towards for their citizens (Adler and Seligman 2016; Exton and Shinwell 2018; Seligman et al. 2011; Huppert and So 2011; McGregor, Camfield, and Woodcock 2009; OECD 2020). While this focus has led to the development of measures of well‐being as a holistic construct, it has not to date included one's sexual and reproductive experiences as a dimension of well‐being. In this commentary, we describe ongoing collaborative work being done to consider how a measure of sexual and reproductive well‐being (SRWB) could be developed for use in diverse global contexts, with the ultimate goal of facilitating a focus on people's lived experiences with sexuality and reproduction as the true guiding star of sexual and reproductive health programs and policies.

## BACKGROUND ON WELL‐BEING AS A MEASURE

Historically, population‐level definitions and measures of social progress were limited to objective economic dimensions, such as gross domestic product (GDP) and per capita income, with the implicit assumption that the national economy, economic growth, and wealth were indicative of the well‐being of a nation. The inadequacy of this approach was identified in economic spheres over the past decades, with increased interest in considering the subjective conceptualization of well‐being—focusing on people's self‐reported evaluation of their own lives, including feeling good, functioning well, and developing one's potential, professional, and personal success (Huppert and So 2011)—that were not captured by standard economic measures.

This shift was exemplified in the 2009 *Report by the Commission on the Measurement of Economic Performance and Social Progress*, which criticized historical economic measures as being weak indicators of social progress and well‐being and called for expanding measures to capture noneconomic aspects of a population's lived experiences (Stiglitz, Sen, and Fitoussi 2014). The conceptualization of well‐being in the report had objective dimensions, such as income, education, and life expectancy, captured by the Human Development Index, and subjective dimensions that reflect how people perceive and experience their life conditions (UNDP 2022). Subsequently, the Organization for Economic Co‐operation and Development (OECD) designed a Framework for Measuring Wellbeing and Progress that includes income, wealth, housing, work, health, skills, environment, safety, subjective well‐being, work‐life balance, social connection, and civic engagement (OECD 2020). This framework is intended to facilitate population‐level measurement of inequalities and progress in achieving well‐being as well as allow for global comparisons of well‐being across nations (OECD 2020).

At the national level, several countries, including Scotland, the Netherlands, the United Arab Emirates, Bhutan, and Australia, have implemented well‐being frameworks and measures to guide and evaluate policy and programmatic outcomes (Cook, Benjamín Kaji, and Davíðsdóttir 2023). For example, the Scottish government's National Performance Framework uses social, environmental, economic, and health indicators to assess progress towards ‘increased well‐being’ (Exton and Shinwell 2018). Ecuador's Buen Vivir (the good life) well‐being framework is a social vision for the future that conceptualizes well‐being across three intersecting levels: the individual, the community, and the environment to facilitate and inform policy development and its evaluation (Mero‐Figeuroa et al. 2020). Bhutan's Gross National Happiness Index also includes measures of psychological well‐being, good governance, and cultural preservation, in addition to common social, environmental, economic, and health measures, resulting in a unique set of indicators by which to quantify sustainable human development and progress that is of increasing interest to neighboring countries and international organizations (Munro 2016; Bhattacharyya, Roy Burman, and Paul 2019).

## WELL‐BEING'S RELATIONSHIP TO SEXUAL AND REPRODUCTIVE HEALTH

Prioritizing and measuring well‐being is, therefore, increasingly commonplace in global and national policy agendas, with well‐being measures being used to assess the success of policies and programs and whether people (and populations) are satisfied with their circumstances and opportunities. However, relatively little attention has been paid to how to incorporate consideration of people's sexuality and reproduction into these measures. Existing conceptualizations of subjective well‐being generally include health, social relationships, and family but not the sexual and reproductive experiences that profoundly influence people's health and social and familial lives. One exception was a study in Belgium that included a question on “sexual life” in a survey assessing subjective well‐being along the domains of health, leisure, family life, and social life. This analysis found that, in addition to sexual well‐being being appropriate for inclusion from a psychometric perspective, an item assessing sexual well‐being improved understanding of overall well‐being (Hooghe 2011).

Similarly, in the sexual and reproductive health field, there has been relative neglect of well‐being. The most common set of metrics aligns to some extent with the concept of well‐being—in that they are focused on people's lived experiences—are measures of quality of life or the psychological experiences with conditions related to sexuality and reproduction. Examples include measures of stress or depression related to infertility and measures of quality of life associated with menstruation, endometriosis, and fibroids (Kitchen et al. 2017; Cassioli et al. 2023; Coyne et al. 2019; Jones et al. 2001). These metrics are important contributions to research and clinical care for these diagnoses, as they enable attention to how people are experiencing these conditions rather than only to clinical assessment of disease burden and impact. However, these metrics remain deficit‐framed in assessing the experience of negative psychological or physical symptoms and focus on narrow aspects of experience rather than a holistic view and therefore are not fully aligned with a well‐being focus.

Research and advocacy related to sexuality has, on the other hand, taken a more transformational approach in the direction of a positive and holistic lens through the elaboration of frameworks related to sexual functioning and pleasure (Werner, Borgmann, and Laan 2023; Mitchell et al. 2021). Related measures like the Canadian Sexual Health Indicators survey and Sexual Wellbeing measures capture more nuanced dimensions of people's lived sexual experiences, such as sexual self‐esteem, satisfaction with sexual experiences, and self‐perceptions of agency and autonomy within decision‐making (Smylie et al. 2013; Rosen et al. 2009; Mitchell et al. 2023). These measures represent a critical step forward in the conceptualization of positive sexuality as being more than the absence of disease and nonconsensual sexual experiences.

In addition to this work on sexual well‐being, there has been substantial recent work on other measures that are positively framed and aligned with a rights‐based approach to sexual and reproductive health. These include measures of reproductive agency and autonomy (Hinson et al. 2019; Upadhyay et al. 2014), experiences of reproductive health care (Holt et al. 2023b), and preference‐aligned fertility management (Holt et al. 2025). These measures are critical tools in efforts to recenter reproductive programs and policy on people's lived experiences and rights. However, they are distinct from well‐being measures in focusing on specific components of people's sexual and reproductive lives and experiences, as opposed to a holistic assessment of their well‐being.

## MOVING TOWARDS A MEASURE OF SEXUAL AND REPRODUCTIVE WELL‐BEING

While there has been substantial improvement in the types of measures that are available in the field of sexual and reproductive health, to date, there has not been an effort to develop a holistic measure of SRWB, focusing not only on sexuality but also on the interconnected experience of reproduction. In addition, existing measures of well‐being pay inadequate attention to sexuality and reproduction. There is, therefore, a need for a concise measure of SRWB focused on the degree to which people achieve positive sexual and reproductive experiences as they themselves define them. This measure could be integrated into existing data collection efforts on national and subnational levels to allow for epidemiological tracking of the extent to which structures and systems enable optimal sexual and reproductive experiences on a population level. Further, such a measure would allow for monitoring specific interventions' impact on this outcome and inform funding, planning, implementation, and evaluation of person‐centered and rights‐based programs. The use of this measure can serve as a counter to efforts to stigmatize and decenter rights in sexual and reproductive health. This measure would align with and reinforce the growing emphasis on well‐being in economic and other spaces and ensure that sexual and reproductive health is not de‐emphasized in the well‐being conversation.

**FIGURE 1:**
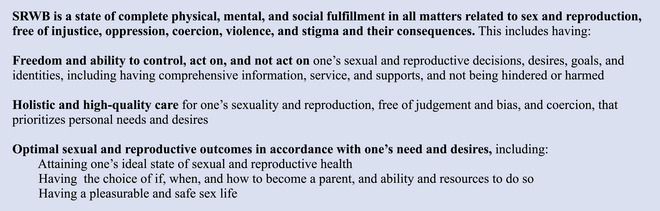
Definition of sexual and reproductive well‐being

To capitalize on this opportunity, in 2021, researchers and advocates initiated an effort to define and measure SRWB in both the United States and in the international context. As described in a recent commentary (Dehlendorf et al. 2025), this work initially focused on developing an overarching definition of SRWB in the United States through a collaborative process with multidisciplinary thought leaders (Figure 1). Recognizing the need to intentionally consider how and if a measure of SRWB could be of use in diverse global contexts and to not assume that work from the United States is relevant outside that context, the Person‐Centered Reproductive Health Program at the University of California San Francisco and the Coalition to Expand Contraceptive Access convened a multidisciplinary group of international experts in sexual and reproductive health in London over two days in 2023. This meeting enabled a collaborative process to consider the potential application of a framework and measure of SRWB to global policy and practice and to elaborate a process for beginning to develop such a measure. The authors of this commentary were participants and thought partners in this meeting and the ongoing engagement that followed. They included representation from Pakistan, India, the United Kingdom, Ethiopia, the United States, Belgium, and The Netherlands.

This group first considered the initial definition of SRWB developed through US‐focused work and its applicability to diverse global contexts. While recognizing the need for ongoing co‐creation and community engagement to arrive at an operational definition for use outside the United States, we concluded that this definition was an appropriate foundation for the global development of an SRWB measure, with broad pertinence due to its focus on universal human experiences related to sexuality and reproduction. Additional qualitative work in diverse contexts and with diverse communities was identified as an essential next step to gain further insight into how to ensure the general applicability of the definition and support its operationalization into a measure.

In working to define a pathway towards developing and operationalizing such a global measure, we identified key considerations that would be critical for the success and impact of this work. First, it is critical to recognize and engage with the fact that measuring development, though scientific, is not apolitical. Discussion of sexuality and reproduction is inherently culturally and politically complex, particularly when working across geographies and communities defined by differing cultural, social, and religious norms (Blystad et al. 2020). Moralizing around sex, sexuality, and reproduction can act as a barrier to the adoption of certain ideas and therefore demands that we pay attention to the socialization of SRWB to facilitate ultimate adoption and dissemination. For example, early in the process, and as part of measure development, we should raise knowledge and awareness about SRWB, including through promotion by highly respected individuals within their social and professional networks and/or creating a “market” for this approach to measurement. Focusing on the particularities of sexual and reproductive politics at an early stage of measure development through thoughtful socialization can help to address any potential resistance and advance its inclusion in policy and programming measurement. Efforts must be taken to meaningfully navigate preexisting sociocultural norms surrounding sex, sexuality, and reproduction and engage relevant technical capacity, resources, and solidarities. In addition, the potential for resistance to the inclusion of sexuality and sexual pleasure in the definition of the measure led us to acknowledge the possibility of having to develop a version of the measure in which only reproductive well‐being is addressed for use in some contexts.

An additional consideration is that a shift towards SRWB requires intentional engagement with individuals and communities with lived experiences to inform the measure creation process, as is increasingly recognized as a best practice in measurement development, in order to move away from traditional methodologies with implicit power dynamics. Drawing on person‐centered care, we need to build measures that focus on the sexual and reproductive aspirations, preferences, and experiences of individuals (Dehlendorf et al. 2016; Dehlendorf et al. 2021). How this translates into measurement development means placing personal experience and community engagement at the center of defining and operationalizing the construct we want to capture, and into the process by which we develop and validate it. Given the sensitive nature of sexuality and reproduction, deeply engaging with individuals and communities can support the development of a measure that truly captures people's lived experiences. This includes ensuring that the questions and answer options are optimized to allow for accurate and honest responses, recognizing the potential for stigma and social norms to interfere with such transparency. This work can draw on the principles of co‐creation, which embrace the collaboration of those who experience a phenomenon at all stages of the research process and ensure that engaging in the process has value for those who participate (Singh et al. 2023). In addition to compensation for time and effort, benefits can include developing awareness of sexual and reproductive health and rights and the promotion of community dialogue on these issues. Importantly, this requires not simply a one‐off engagement but involves continuous and ongoing dialogue with diverse communities (Singh et al. 2023; Vargas et al. 2022). A more person‐ and experience‐centric research process can improve the alignment of the researchers’ aims and people's needs and draw on knowledge in its many forms to generate new and innovative ideas (Bird et al. 2021; Greenhalgh et al. 2019). Such intensive engagement with the community and others influenced by the measure has the added benefit of contributing to creating awareness of SRWB to facilitate its long‐term adoption and use.

An additional consideration raised in this meeting related to the benefits and potential limitations of developing a measure designed to capture people's subjective assessment of their SRWB. While people's opinions about their lives are of critical importance, there is also the potential limitation that people who do not have high expectations for their experiences may answer positively, even if they do not have what we would “objectively” consider a positive experience. This is linked to the concept of critical consciousness and people's ability (or inability) to recognize and act against oppressive forces in their lives (Kabeer 1994). Recognizing this prompted discussion of the need to contextualize any measure of SRWB in light of other socioecological factors, such as gender empowerment and social inclusion. Explicit guidance about this contextualization would protect against the potential for positive SRWB scores to be interpreted in isolation without consideration of how people's subjectivities are embedded in external environments.

The group also recognized the need to acknowledge the tension between creating a measure that can be meaningful and valid across geographies, experiences, and contexts, with an awareness of the deeply personal and nuanced experiences of sexuality and reproduction. This is particularly relevant when considering the importance of being inclusive of the experiences of those whose sexuality and reproduction are stigmatized, devalued, or otherwise oppressed or made invisible. While there is a clear benefit to creating a measure that can be used, tracked, and compared broadly, it is critical to simultaneously explore the need for additional and more nuanced measures that capture specific experiences—such as whether someone experiencing childbirth experienced respectful maternity care or someone with a gender‐expansive identity was able to receive gender‐affirming care. Additional measures can be generated to capture the more nuanced experiences in different contexts, such as more detailed evaluations of sexuality in countries where gender inequities and/or religious conservatives are more prominent. Ultimately, our goal is to create a succinct measure of SRWB) using general themes related to universal experiences and preferences, such as experiences of sexual and reproductive health care and achievement of one's fertility preferences, whatever they may be. We also seek to appreciate the broader ecosystem of measures that can and should supplement this broad measure through a more detailed assessment. Together, this effort can provide the focus on and ability to measure SRWB on a global, national, and subnational level while providing additional, actionable insights into barriers to achieving the highest possible level of well‐being across contexts and experiences.

Finally, the group considered the socialization and implementation challenges of the measure once it was created. Barriers to broad acceptance and uptake of this measure that were identified included the potential for resistance from anti‐gender movements, the reality of indicator and measurement fatigue, with limited space for additional questions on existing data collection platforms, and resistance or inertia on the part of governments and systems due to the aspirational and holistic (and therefore less immediately actionable) nature of the measure. Strategies for engaging with diverse sectors across geographies and sectors to promote awareness and acceptability were discussed, including having an advisory group throughout the measure development process and engaging with both grassroots and governments/funders to socialize the measure.

## NEXT STEPS TO DEVELOP A MEASURE OF SEXUAL AND REPRODUCTIVE WELL‐BEING

There is a clear opportunity to develop a measure that assesses, on an individual‐ and population‐level, the degree to which individuals have achieved the sexual and reproductive lives they wish to have. The inputs and insights from diverse global sexual and reproductive health thought leaders described above have provided critical guidance as we develop the SRWB measure to meet this opportunity. This work will include intensive qualitative work in multiple geographies to test and optimize the definition of SRWB in order to enhance its relevance and generalizability and develop items that capture aspects of the definition. Following the development of a draft instrument, we plan to conduct initial cognitive testing of the items, additional cognitive testing in diverse contexts, and then proceed with qualitative and quantitative testing to inform the delineation of the final measure using psychometric techniques and ongoing engagement with communities and stakeholders. Ultimately, we desire to create a measure that is scientifically and conceptually valid and feasible for use in international, national, and subnational contexts and initiatives, ranging from Demographic and Health Surveys and the next rendition of the Sustainable Development goals to programmatic and policy evaluations.

Recognizing the sensitive nature of sexuality and reproduction and the challenges entailed in striving to develop a universal and generalizable measure of SRWB, we welcome inputs and feedback from those in the sexual and reproductive health sphere, as well as those with experience and expertise in the measurement of well‐being, to contribute in formal and informal ways to this process.

## CONCLUSION

Through building on the increasing attention to well‐being in economics and policy discussions, the development of a measure of SRWB will provide a mechanism to draw attention to and monitor the extent to which people are having positive and meaningful sexual and reproductive experiences. Leveraging initial work being done in the United States to develop a definition and measurement framework, we have the opportunity to build towards a world where success in sexual and reproductive health is not defined by measures of adverse outcomes but rather by whether people can achieve the sexual and reproductive lives they wish to have.
